# Dragon’s Blood as a promising strategy against cerebral ischemia-induced memory processing impairment

**DOI:** 10.3389/fncel.2026.1960961

**Published:** 2026-10-26

**Authors:** Pinqiu Chen, Jingjing Zhang, Xiangli Yao

**Affiliations:** Shijiazhuang Medical College, Shijiazhuang, Hebei, China

**Keywords:** Dragon’s Blood, hippocampus, memory dysfunction, neuroinflammation, post-stroke cognitive impairment, prefrontal cortex

## Abstract

Cerebral ischemia frequently triggers persistent learning and memory dysfunction, which is closely associated with structural and functional impairments of memory-related brain regions including the hippocampus, amygdala and cerebral cortex. Disrupted oxidative homeostasis, excessive neuroinflammation, synaptic degeneration and imbalanced neurotransmitter signaling jointly constitute cascaded pathological processes that destroy the integrity of hippocampus-amygdala-prefrontal cortex circuit. Dragon’s Blood, a traditional herbal medicine with multiple bioactive constituents, exhibits prominent antioxidant, anti-inflammatory and neuroprotective properties. Accumulating evidence indicates that Dragon’s Blood and its major active components (loureirins and pterostilbene) can alleviate ischemic neuronal injury, stabilize synaptic structure, restore the balance of multiple neurotransmitter systems, and reconstruct damaged memory circuits. This review systematically summarizes the multi-target mechanisms by which Dragon’s Blood ameliorates cerebral ischemia-induced memory impairment, focusing on its regulatory effects on oxidative stress, microglial polarization, synaptic plasticity and interregional neural circuits. We further discuss existing limitations of current research and prospective directions for subsequent preclinical and translational investigations. This work aims to provide theoretical references for developing natural compound-based interventions against post-stroke cognitive disorders.

## 1 Introduction

Stroke ranks as the second leading cause of death and the third leading cause of disability worldwide, with ischemic stroke predominating (Naghavi et al., 2025; Wu et al., 2026; Sharma and Lee, 2025; Wang et al., 2016). Cerebral ischemia–reperfusion injury is a multi-mechanism secondary injury triggered after the restoration of cerebral blood flow. Survivors often have residual functional impairments in movement, cognition, and speech. Among these, post-stroke cognitive impairment (PSCI) is a common disabling sequela, with a global incidence of 20–80%; approximately 60% of patients may develop cognitive decline within 1 year of stroke onset (Guan et al., 2026; Dowling et al., 2024; Hu and Chen, 2017; Filler et al., 2025). Previous studies have shown that PSCI is significantly associated with apoplexy relapse and all-cause death risk (Moroney et al., 1997; Tatemichi et al., 1994). Research has also found that cognitive function in elderly patients with ischemic stroke gradually declines over time (Chen X. et al., 2026). Unlike the physical function impairment caused by stroke, which may gradually improve with rehabilitation, cognitive function in these patients often shows a persistent decline. Ischemic stroke triggers a cascade of pathological events including energy depletion, excitotoxicity, oxidative stress, neuro-inflammation and blood–brain barrier disruption, which collectively contribute to neuronal death and cerebral injury. Such cognitive deficits, owing to their subtlety, are frequently overlooked in clinical settings, severely impacting patients’ quality of life and prognosis. However, memory dysfunction following cerebral ischemia remains a major clinical challenge for post-stroke cognitive impairment.

Neuroimaging studies have shown that infarcts in key brain regions play a significant role in the risk of PSCI. These lesions include infarcts in the dominant thalamus, the angular gyrus, the deep frontal lobe, the medial temporal lobe, the hippocampus, and the left hemisphere, as well as multiple infarcts in both hemispheres (Weaver et al., 2021; Vermeer et al., 2003). The hippocampus is a critical brain region that regulates memory function and plays a vital role in the encoding and consolidation of short- and long-term memory, as well as in spatial processing (Huang et al., 2026). Patients who have suffered a stroke exhibit varying degrees of impairment in hippocampal function. Clinical studies have confirmed that even when patients experience isolated infarction in the hippocampal perfusion zone without accompanying extensive cortical ischemia, they may still exhibit persistent impairments in episodic memory and learning ability. Ischemia in different sub-regions of the hippocampus can cause selective impairment of specific memory subtypes, and in severe cases, this may progress to hippocampal dementia (Kumral et al., 2015), fully confirming that the hippocampus is a key brain region mediating post-ischemic memory dysfunction. Resting-state functional magnetic resonance imaging (fMRI) studies have further revealed that patients with ischemic stroke and cognitive impairment exhibit disrupted hippocampal functional connectivity patterns, with impaired circuit function between the hippocampus and memory-related brain regions such as the prefrontal cortex; moreover, the degree of reduced functional connectivity is closely correlated with the severity of memory deficits (Jung et al., 2021). Functional near-infrared spectroscopy (fNIRS) studies have similarly confirmed that patients with post-stroke cognitive impairment exhibit abnormalities in oxygen consumption in the prefrontal cortex, and the degree of impairment in cortical neural activity can be used to stratify the severity of cognitive impairment (Zhang et al., 2025).

Cognitive impairment caused by cerebral ischemia exhibits marked regional selectivity; not all cerebral infarcts lead to memory decline. Only ischemic lesions that damage key “strategic sites” within memory circuits are more likely to result in persistent memory impairment. A multivariate lesion-symptom mapping study indicates that brain regions involved in memory encoding and information transmission—such as the thalamus, the medial temporal lobe hippocampus, the caudate nucleus, and the anterior radiate body—constitute critical cognitive hubs; when ischemic infarcts occur in these regions, even small lesions can disrupt information transmission within the memory neural network, ultimately leading to significant memory impairment (Zhao et al., 2018). In animal models, cerebral ischemia can induce significant declines in learning and memory behaviors, manifesting as multidimensional cognitive impairments in spatial memory, working memory, and recognition memory (Schmidt et al., 2015). Although significant advances have been made in revascularization techniques in recent years, the incidence of PSCI has not decreased accordingly (Balcerzak et al., 2026; Ettelt et al., 2020). Therefore, investigating the mechanisms by which cerebral ischemia damages core brain regions associated with memory will help elucidate the pathogenesis of post-ischemic memory impairments. Currently, this field still faces challenges such as insufficient elucidation of underlying mechanisms, a lack of early assessment tools, and a scarcity of effective therapeutic agents. There is an urgent need to identify candidate drugs capable of blocking the pathological cascade of ischemia through multiple targets and protecting memory circuits. The traditional herbal medicine Dragon’s Blood possesses multiple activities, including antioxidant, anti-inflammatory, and neuroprotective effects. By regulating multiple damage pathways and maintaining the homeostasis of cognitive circuits, it can improve the decline in learning and memory functions caused by cerebral ischemia. A systematic summary of its underlying mechanisms is expected to provide new strategies for the intervention of ischemic cognitive impairment.

## 2 Key bioactive constituents of Dragon’s Blood against memory impairment

The traditional source of Dragon’s Blood resin is plants of the genus Dracaena, which also serve as the most fundamental botanical source of this medicinal material (Xin et al., 2024). Currently, the recognized tree species from which Dragon’s Blood resin is derived include *Dracaena cinnabari* Balf.f. (Socotra Dragon’s Blood), *Dracaena draco* (L.) L. (African Dragon’s Blood), *Dracaena cochinchinensis* and *Dracaena cambodiana* (Hainan Dragon’s Blood), among others (Zhang Z. et al., 2022). Among these, wild populations of *Dracaena cinnabari* Balf.f. and *Dracaena draco* (L.) L. are scarce and have been listed by the IUCN as Vulnerable and Endangered species, respectively. In China, medicinal Dragon’s Blood is a resin obtained through the extraction and processing of the resinous wood of *Dracaena cochinchinensis* (Lour.) S. C. Chen, a species of the genus *Dracaena* in the Asparagaceae family (Chen et al., 2024). It has been used since ancient times as a dye and rust-preventive coating, possessing both economic value and traditional applications (Sohlman, 2004). Modern pharmacology indicates that Dragon’s Blood resin exhibits a wide range of pharmacological activities, including antioxidant stress, anti-inflammatory, antithrombotic/antiplatelet aggregation, blood–brain barrier protection, and antitumor effects, and has received significant attention from scholars both domestically and internationally (De Albuquerque et al., 2025). Nevertheless, it should be noted that “Dragon’s Blood” is a general term for red resinous substances obtained from multiple plant taxa. In this review, we exclusively focus on *Dracaena cochinchinensis*-derived Resina Draconis. Other botanical sources of “Dragon’s Blood” and their preparations are only briefly mentioned as contextual background and are not within the core scope of this work. Given the substantial differences in chemical profiles among dragon’s-blood materials from distinct plant origins, pharmacological findings obtained from one source should not be extrapolated to other chemically distinct resins.

Existing research indicates that Dragon’s Blood primarily contains phenolic, terpenoid, and steroid compounds, with phenolics being its signature active components (Asmara et al., 2026). Terpenes and steroids such as 4-ene-3-cholest-3-en-11-one, cholesterol, and stigmasterol, as well as more than 180 types of phenolic compounds, have been isolated from plants of the genus Dracaena (Sun et al., 2019b); among which flavonoids and diphenylstyrenes are present in the highest concentrations; flavonoids account for 50–80% of the total constituents and include chalcones, dihydrochalcones, high-isoflavones, high-isoflavanones, anthocyanins, flavones and flavanols, flavanols, flavonoid dimers, flavonoid trimers, and dihydroflavones, with Dragon’s Blood resins A, B, C, and D serving as typical representatives (Ma W. et al., 2026). Recent studies have also identified dihydrochalcane-ring-fused trimers with anti-inflammatory activity in Dragon’s Blood resin plants (Pang et al., 2019). The chemical structures of the major bioactive monomers (compounds 1–5), namely Loureirin A (1), Loureirin B (2), 7,4′-dihydroxyflavone (3), resveratrol (4), and pterostilbene (5), are illustrated in Figure 1, and their relevant information is summarized in Table 1. These compounds possess multiple pharmacological properties, including anti-dementia, antioxidant, anti-inflammatory, neuroprotective, antitumor, and antithrombotic effects (Lang et al., 2020; Yan and Zhao, 2025).

**Figure 1:**
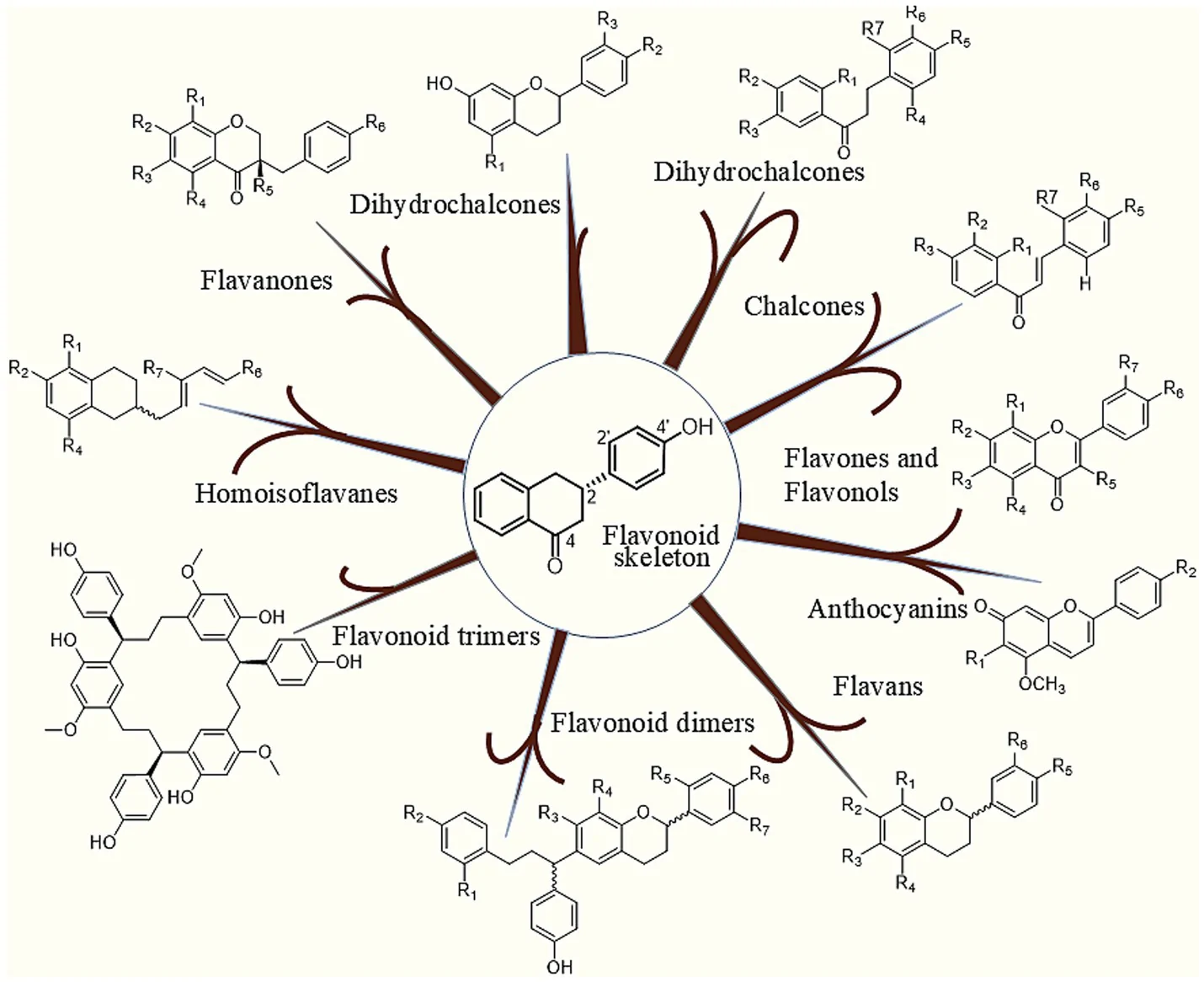
Major active constituents of Dragon’s Blood.

**Table 1:** Major active ingredients of dragon’s blood and their pharmacological effects on neurodegenerative diseases.

Active ingredient	Structure	Pharmacological effects	Indicators	References
Loureirin A (C_17_H_18_O_4_)	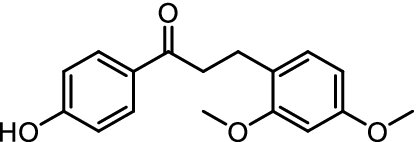	Antioxidant; BBB protection; antithrombotic	MDA↓, SOD↑, CAT↑, GSH-Px↑; AQP4↓; p-Akt↓, PI3K/AKT signaling↓	Yang et al. (2025); Zhou et al. (2015)
Loureirin B (C_18_H_20_O_5_)	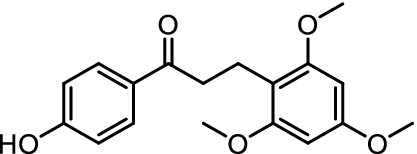	Antioxidation; anti-inflammation; anticoagulation; antithrombosis	Nrf2↑, HO-1↑, Bcl-2↑; TLR4 mRNA↓, p-NF-κB p65↓; xCT mRNA↑; PAI-1/uPA complex↓	Jiang et al. (2020); Zhou et al. (2015); He et al. (2017)
Loureirin C (C₁₆H₁₆O₄)	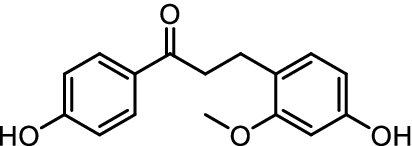	Anti-ferroptotic neuroprotection via Nrf2; enhances cognition.	Nrf2, GPX4, SYP, PSD95, GAP43, neuronal count, cognition tests	Liu et al. (2023a)
7,4′-dihydroxyflavone (C_15_H_10_O_4_)	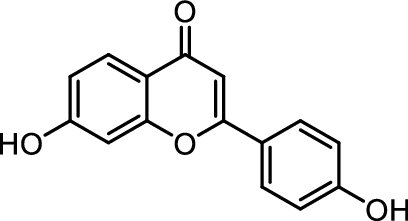	Anti-inflammation	iNOS mRNA↓, TNF-α↓, IL-6↓, NO↓	Pan et al. (2018)
Pterostilbene (C_16_H_16_O_3_)	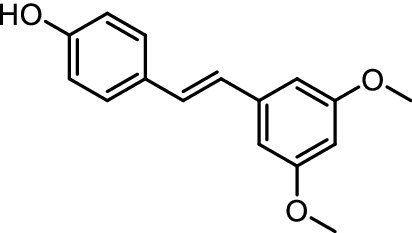	Antioxidant, anti-inflammatory, neuroprotective, BBB-protective	NLRP3↓, LC3-II↑, ROS↓, CAT↑, GSH↑; p-NF-κB↓, COX-2↓, iNOS↓, MMPs↓; TNF-α↓, IL-1β↓, IL-6↓; Bax/Bcl-2↓, Na^+^↓	Dutta et al. (2025); Wang et al. (2015); Yang et al. (2022); He et al. (2017)
Resveratrol (C_14_H_12_O_3_)	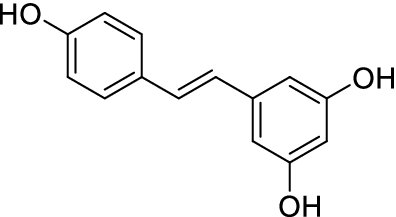	Antiferroptotic; antioxidant, anti-inflammatory, neuroprotective, BBB-protective	Nrf2/HO-1↑, MDA↓; GPx/SOD/CAT↑; TNF-α/IL-1β/NLRP3↓, IL-10↑; Bax/Bcl-2↓, caspase-3↓; p-Akt/GSK-3β↑; ICAM-1/VCAM-1↓	Xin et al. (2011); Liang et al. (2021); Heo et al. (2010); Wei et al. (2015)

↑ indicates increased expression/activity; ↓ indicates decreased expression/activity. MDA, malondialdehyde; SOD, superoxide dismutase; CAT, catalase; GSH-Px, glutathione peroxidase; AQP4, aquaporin 4; GPX4, glutathione peroxidase 4; SYP, synaptophysin; PSD95, postsynaptic density protein 95; GAP43, growth-associated protein 43; iNOS, inducible nitric oxide synthase; TNF-α, tumor necrosis factor-α; IL, interleukin; NLRP3, NLR family pyrin domain containing 3; ROS, reactive oxygen species; COX-2, cyclooxygenase-2; MMPs, matrix metalloproteinases; ICAM-1, intercellular adhesion molecule-1; VCAM-1, vascular cell adhesion molecule-1.

With Dragon’s Blood glycosides A/B/C, Pterostilbene, and Dragon’s Blood glycoside as the core active ingredients, this compound provides a multi-component synergistic intervention for post-stroke memory impairment. The signature flavonoid components, Dragon’s Blood flavonoids A, B, and C, play a particularly critical role: by scavenging reactive oxygen species (ROS) in the brain and upregulating antioxidant pathways to alleviate oxidative stress, while simultaneously inhibiting abnormal microglial activation to block neuroinflammation, they reduce damage to hippocampal neurons and maintain neuronal homeostasis, ultimately improving impairments in memory formation and retrieval (De Albuquerque et al., 2025; Li R. et al., 2023; Jiang et al., 2020). Behavioral experiments using the Morris water maze and Y-maze in mouse models of vascular dementia have confirmed that Loureirin C significantly enhances spatial and working memory in animals, upregulates the expression of synaptic-related proteins in the hippocampus, and repairs damaged neurons; simultaneously, Loureirin C shows potential for treating Alzheimer’s disease, Parkinson’s disease, vascular dementia, and depression accompanied by cognitive decline (Zou et al., 2017); Dragon’s Blood Resin B exerts antioxidant and anti-inflammatory effects through multiple signaling pathways, thereby alleviating damage to hippocampal neurons (Li R. et al., 2023); Dragon’s Blood Resins A and B can also antagonize the neurotoxicity of β-amyloid, further protecting neural function (Ospondpant et al., 2022).

Loureirin D can antagonize the binding of adenosine diphosphate (ADP) to the P2Y12 receptor, inhibit platelet activation and adhesion, and reduce the release of prothrombotic factors such as ADP, thereby exerting a significant antithrombotic effect (Peng et al., 2026).

Pterostilbene, a highly fat-soluble diphenylethylene compound found in Pterostilbene, has excellent permeability across the blood–brain barrier (BBB) and can target brain tissue. By regulating neural plasticity pathways, it promotes hippocampal neurogenesis and synaptic repair, thereby improving impairments in memory consolidation and retrieval caused by neurological damage (Yang et al., 2019). Recent high-impact research has demonstrated that Pterostilbene is beneficial for the treatment of certain neurological disorders, including neurodegenerative diseases, learning and memory impairments, and cerebral ischemia (Leong, 2026). Compared to resveratrol, its dimethoxy structure enhances lipophilicity and brain accumulation, upregulates synaptic proteins in the hippocampus, increases dendritic spines, and improves memory through dual pathways of neurogenesis and synaptic plasticity (Yang et al., 2019; Meng et al., 2019).

In addition, secondary active components in Dragon’s Blood resin—such as quercetin, kaempferol, and β-sitosterol—work synergistically to further enhance its overall memory-protective effects by supporting antioxidant and anti-inflammatory actions and stabilizing the structure of nerve cell membranes; these are indispensable auxiliary active substances for alleviating memory impairment (Ning et al., 2025; Wang et al., 2025; Mughal et al., 2026).

Furthermore, existing pre-clinical studies have shown that the dichloromethane extract and characteristic isoflavan- and isoflavone-type compounds from Dragon’s Blood can target quinone oxidoreductase 1, inhibit acetylcholinesterase activity, and suppress amyloid aggregation. These constituents can mitigate neurodegeneration-associated damage, suggesting potential value for ameliorating memory deficits following cerebral ischaemia (Tang et al., 2019; Noshita et al., 2021; Li et al., 2014).

## 3 Mechanisms underlying Dragon’s Blood-mediated memory improvement

### 3.1 Neuroprotective regulation of Dragon’s Blood on memory-associated brain regions in cerebral ischemia

#### 3.1.1 Protection of the hippocampus

Neuroinflammation, oxidative stress, and massive neuronal apoptosis triggered by cerebral ischemia are the underlying causes of neurotransmitter imbalance, disruption of hippocampal circuits, and cognitive and memory decline (Yu et al., 2025; Zhou et al., 2026). Studies have confirmed that the hippocampus is the primary region responsible for learning and memory, and damage to hippocampal neurons following cerebral ischemia is the main mechanism underlying post-ischemic memory impairment (Banquet et al., 2021). Cerebral ischemia can lead to a massive accumulation of reactive oxygen species (ROS) in the body and downregulation of antioxidant enzyme activity. This is accompanied by energy metabolism imbalances caused by ATP deficiency, which in turn exacerbates oxidative stress damage; it results in reduced neuronal apoptosis in the hippocampal CA1, CA3, and dentate gyrus regions, disruption of mitochondrial structure, and abnormalities in synaptic morphology and number, thereby inhibiting synaptic long-term potentiation and ultimately leading to impaired spatial learning and memory function (Lana et al., 2020; Sadeghzadeh et al., 2023). In addition to static structural and molecular damage, recent studies have further revealed that ischemia-induced neurotransmitter imbalances and disruption of hippocampal circuits manifest at the systemic level as accelerated transitions between dynamic abnormal states—known as “hidden brain states”—and prolonged abnormal network dwell times in PSCI, which are highly correlated with cognitive decline (Ma C. et al., 2026).

Active components in Dragon’s Blood resin—such as Dragon’s Blood resins A, B, and C, and Pterostilbene—can target hippocampal tissue. By inhibiting oxidative stress, improving mitochondrial dysfunction, and blocking inflammatory cascades, they intervene upstream in the pathological damage process caused by cerebral ischemia, exerting a neuroprotective effect and thereby improving ischemia-induced learning and memory impairments. Studies have shown that total flavonoids from Dragon’s Blood resin can significantly improve pathological damage to hippocampal neurons in rats with cerebral ischemia–reperfusion, reduce neuronal apoptosis, and simultaneously enhance spatial learning and memory abilities. The mechanism underlying these effects is closely related to the reduction of oxidative stress in the hippocampus and the enhancement of antioxidant enzyme activity (Zhu et al., 2025). Loureirin A (from *Dracaena cochinchinensis*) can reduce cerebral ischemic infarct volume, improve neuronal morphology in the hippocampal CA1 region, enhance antioxidant enzyme activities, inhibit neuronal apoptosis, and improve learning and memory functions (Yang et al., 2010). Further studies have found that Dragon’s Blood can reduce neurological deficit scores in rats with cerebral ischemia, decrease cerebral edema and infarct volume, increase SOD and GSH-Px activity in hippocampal tissue, lower MDA levels, and improve spatial learning and memory abilities (Zhang X. et al., 2022). A 50% ethanol extract of Dragon’s Blood (50 μg/mL) can significantly reduce the Bax/Bcl-2 ratio (Ran et al., 2016), increase the expression of brain-derived neurotrophic factor, and decrease the expression of pro-apoptotic caspase-3, thereby inhibiting apoptosis in rat hippocampal neurons induced by ^60^Co-γ radiation and exerting a protective effect against neuronal damage (Xin et al., 2012). *In vivo* experiments have confirmed that Dragon’s Blood resin can significantly alleviate ischemia-induced hippocampal neuronal damage by inhibiting oxidative stress and mitochondrial dysfunction, upregulate the expression of proteins associated with synaptic plasticity, and ultimately improve learning and memory functions (Xu et al., 2025).

Loureirin C exhibits estrogenic activity (Xu et al., 2023) and exerts neuroprotective effects (Chen et al., 2025). It can directly scavenge ROS, inhibit mitochondrial ROS production, and alleviate oxidative stress and DNA damage in the hippocampus (Liu et al., 2023a); studies have confirmed that Loureirin C (from *Dracaena cochinchinensis*) exerts neuroprotective effects by inhibiting ferroptosis through regulation of the Nrf2 pathway (Liu et al., 2023a). It upregulates synapse-related proteins SYP/PSD95/GAP43 and improves hippocampal synaptic structure and function; it reduces hippocampal neuron loss, improves ischemic learning and memory impairments, and has been demonstrated in patents to be useful for the preparation of drugs for the prevention and treatment of vascular dementia, Alzheimer’s disease, Parkinson’s disease, and depression, as well as for improving cognitive and emotional abnormalities in animal models (Zou et al., 2017).

Pterostilbene can cross the BBB and accumulate in the hippocampus (Dutta et al., 2025), exerting its effects by intervening in the upstream pathological cascade of cerebral ischemia through multiple pathways. On the one hand, it activates the Nrf2/ARE pathway, scavenges ROS, and alleviates oxidative stress-induced damage in the hippocampus; on the other hand, it improves mitochondrial energy metabolism and mitigates ultrastructural damage in the hippocampal CA1 and DG regions (Hu et al., 2011; Tian et al., 2026); simultaneously, it inhibits the COX-2/PGD₂/DP₂ signaling pathway and the release of inflammatory factors such as IL-1β and TNF-α, thereby blocking the hippocampal neuroinflammatory cascade, which in turn reduces neuronal apoptosis and improves ischemia-induced learning and memory impairments (Yang et al., 2024; Yan et al., 2021). This study investigated the protective effects of different concentrations of Pterostilbene (1.25 μmol/L, 2.5 μmol/L, 5 μmol/L) against OGD/R-induced damage in mouse hippocampal neurons (HT22). The results revealed a dose-dependent relationship between neuronal survival rate and Pterostilbene concentration, with cell survival rates at each concentration being (55.45 ± 3.62)%, (67.23 ± 4.37)%, and (81.87 ± 5.02)%, respectively, and Western blot results showed that the Bax/Bcl-2 ratio decreased progressively with increasing concentrations of *Pterocarpus indicus*, suggesting that *Pterocarpus indicus* exerts a certain protective effect against OGD/R-induced damage in neuronal cells (Wang et al., 2015).

Dragon’s Blood and its active components exert a certain protective effect on damaged neurons by counteracting brain ischemia-induced neuronal apoptosis and improving neuronal survival rates.

#### 3.1.2 Protection of the prefrontal cortex

The prefrontal cortex is a core brain region that regulates working memory, memory encoding and retrieval, memory consolidation, and inhibitory control (Li S. et al., 2023). Among these regions, the dorsolateral and medial prefrontal cortices are highly susceptible to cerebral ischemia; this area is primarily supplied by the anterior cerebral artery and the middle cerebral artery. Ischemic damage to brain tissue can induce neuronal apoptosis, ferroptosis, oxidative stress imbalance, mitochondrial dysfunction, and neuroinflammatory responses, leading to the destruction of synaptic structures and resulting in working memory dysfunction. Concurrently, this disrupts the connections within the hippocampal-prefrontal memory neural circuit, further exacerbating cognitive impairment (Barati et al., 2025; Tang et al., 2026).

The primary active ingredient in Dragon’s Blood exerts its effects on the prefrontal cortex, providing neuroprotection through two key mechanisms: inhibiting the excessive activation of microglia and repairing synapses, as well as reshaping the hippocampal-prefrontal memory circuit. Following an ischemic stroke, resting microglia can polarize into pro-inflammatory M1 or anti-inflammatory M2 types under the influence of various signaling pathways, mediating neuroinflammatory damage and neuroprotective repair effects, respectively (Xu et al., 2020) (Figure 2). Loureirin A can inhibit the excessive activation of M1-type microglia in the prefrontal cortex following cerebral ischemia, reduce the release of inflammatory factors, and block the vicious cycle of neuroinflammation (Jiang et al., 2020).

**Figure 2:**
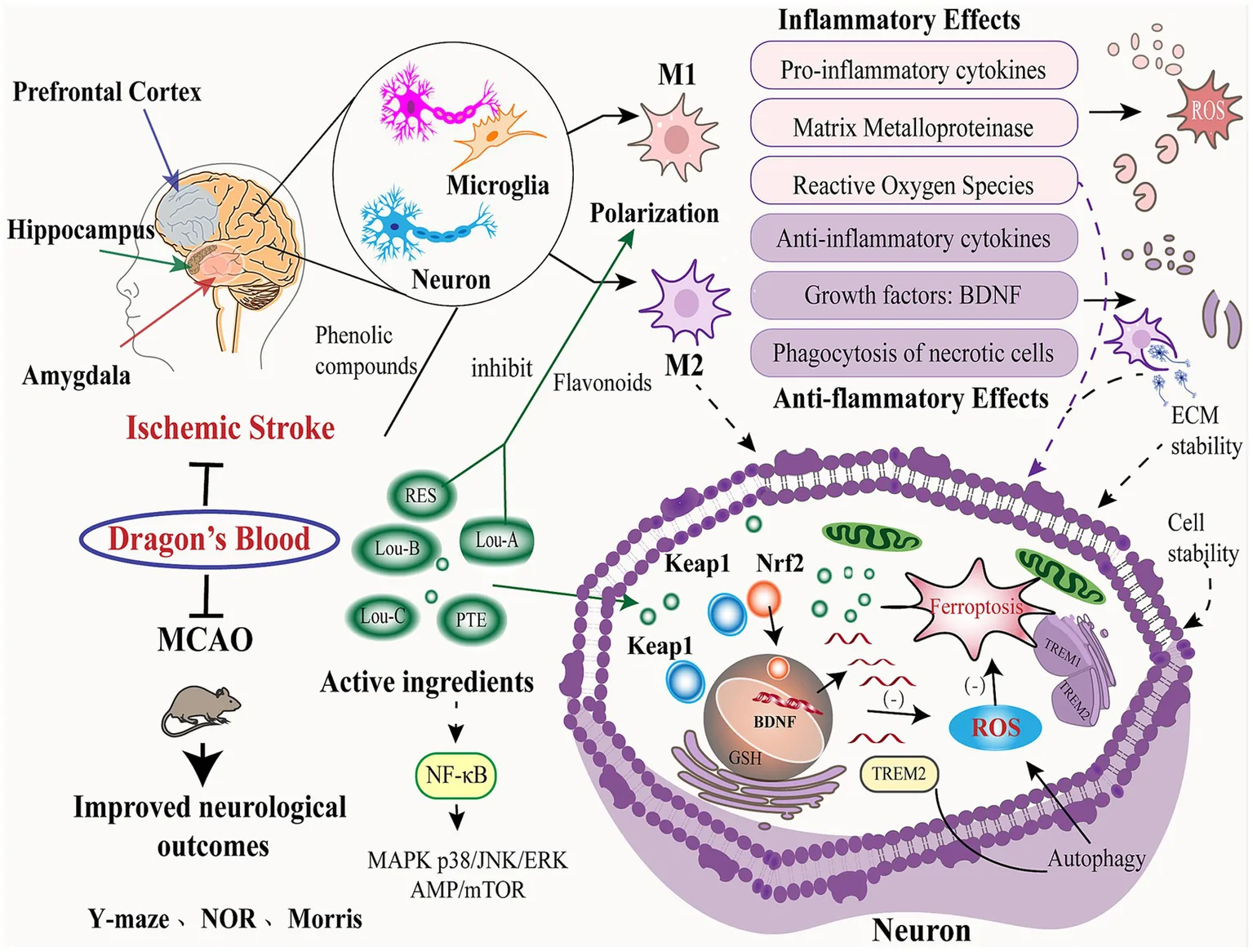
Dragon’s Blood protects against ischemic stroke by regulating microglial polarization and Nrf2-mediated antioxidation. Solid arrows represent direct regulatory effects. MCAO, middle cerebral artery occlusion; NOR, novel object recognition; ROS, reactive oxygen species; BDNF, brain-derived neurotrophic factor. M1 microglia exhibits pro-inflammatory effects, while M2 microglia exerts anti-inflammatory effects. Dragon’s Blood-derived active ingredients modulate microglial M1/M2 polarization, activate the Nrf2/Keap1 pathway, suppress NF-κB-MAPK-AMPK-mTOR cascades, upregulate BDNF expression, suppress ROS accumulation and neuronal ferroptosis, and ultimately improve neurological and cognitive deficits evaluated by Y-maze, NOR and Morris tests.

Following ischemic stroke, the expression of synapse-related proteins PSD 95 and synaptophysin in the prefrontal cortex and hippocampus is significantly downregulated, synaptic structure is disrupted, and functional connectivity in the hippocampus-prefrontal cortex circuit is weakened (Yang et al., 2025). Pterostilbene significantly upregulates the expression of the presynaptic protein synaptophysin and the postsynaptic protein PSD 95 in the prefrontal cortex and hippocampus of rats in an ischemic model, improves synaptic ultrastructural damage, and—by activating the CREB/BDNF pathway—enhances hippocampal-prefrontal functional connectivity, promotes the reconstruction of memory circuits, and improves spatial learning and memory abilities (Dutta et al., 2026). Loureirin C acts directly on the prefrontal cortex. Through mechanisms such as activating the BDNF/AKT/mTOR/CREB pathway, inhibiting IL-17-mediated neuroinflammation, and selectively regulating ERα-mediated antioxidant activity, it repairs prefrontal cortical synaptic structures, enhances synaptic plasticity, and collaborates with the hippocampus to reconstruct memory circuits (Liu et al., 2023b; Yang et al., 2022).

#### 3.1.3 Protection of the amygdala

Growing evidence demonstrates that the amygdala, particularly its basolateral subregion, cooperates with the hippocampus and prefrontal-cortex to regulate emotional memory formation and consolidation following cerebral ischemia. The amygdala is a key brain region involved in regulating the formation and consolidation of emotional memories and works in concert with the hippocampus and prefrontal cortex to regulate memory functions (Yu et al., 2021); population-based studies have found that patients with mild cognitive impairment exhibit a significant reduction in amygdala volume, which progressively atrophies as cognitive impairment worsens, showing a decreasing trend in volume from healthy individuals to those with mild cognitive impairment to patients with Alzheimer’s disease (Zheng et al., 2023). Atrophy occurs in multiple subregions of the amygdala, most notably in the basolateral nucleus; this region connects the prefrontal cortex with the hippocampus, and its atrophy and degeneration may impair cognitive functions that rely on these connections, such as emotional regulation, learning, and memory Cerebral ischemic injury can cause massive apoptosis of neurons in the basolateral subregion of the amygdala, with a neuronal loss rate of up to 20–25%; it also disrupts synaptic structures, thereby blocking information transmission in the amygdala–hippocampus–prefrontal cortex neural circuit (Cheng et al., 2024).

The above studies suggest that cerebral ischemia can lead to atrophy and functional impairment of the amygdala (particularly the basolateral amygdala, BLA), which is closely associated with cognitive decline. Further morphological analysis revealed region-specific atrophy in various subregions of the amygdala, with the most significant atrophy observed in the BLA. The BLA projects extensively to the frontal lobe, hippocampus, and sensory cortex and is involved in emotional regulation, learning, and memory. Current research on Dragon’s Blood and its active components in cerebral ischemia has primarily focused on the overall protection of the cortex, hippocampus, and limbic system; no reports have yet been published that directly target the BLA as a core target. However, Dragon’s Blood resins A, B, and C, as well as Pterostilbene, can protect neurons in the perifeal and limbic regions following ischemia through mechanisms such as antioxidant and anti-inflammatory effects and the inhibition of ferroptosis. Cumulative evidence indicates pterostilbene activates the BDNF/TrkB axis in cerebral ischemia and amygdalar BDNF/TrkB mediates anxiety responses, suggesting pterostilbene may modulate amygdalar BDNF/TrkB to relieve post-ischemic anxiety, these findings suggest that the active components of Dragon’s Blood may have a potential protective effect on the ischemic BLA, warranting further validation. Cumulative evidence indicates pterostilbene activates the BDNF/TrkB axis in cerebral ischemia (Tian et al., 2026) and amygdalar BDNF/TrkB mediates anxiety responses (Sha et al., 2023). As pterostilbene is one of the major active constituents of Dragon’s Blood, these findings suggest that Dragon’s Blood-derived components may exert potential protective effects on the ischemic BLA, warranting further validation.

### 3.2 Pharmacological mechanisms of Dragon’s Blood in ameliorating ischemia-induced memory impairment

#### 3.2.1 Regulation of oxidative stress

Oxidative stress imbalance induced by cerebral ischemia serves as the central pathological link connecting damage to hippocampal and striatal neurons, neurotransmitter dysregulation, and synaptic degeneration, ultimately leading to impaired memory processing. The accumulation of large amounts of reactive oxygen species triggers a cascade of damage, disrupting the information transmission function of the hippocampus-amygdala-prefrontal cortex circuit. Dragon’s Blood and its active components can scavenge reactive oxygen species, activate the Nrf2 pathway, and inhibit neuroinflammation; they protect neurons and synaptic structures within the circuit, restore the homeostasis of various neurotransmitters, break the vicious cycle of oxidative stress, repair cognitive circuits, and improve memory deficits induced by cerebral ischemia.

In recent years, numerous studies have reported that Dragon’s Blood and its major active components exhibit beneficial effects in combating oxidative stress. Compounds such as sandalwood lactone, resveratrol, Loureirin B, and Loureirin A can protect brain tissue by mitigating oxidative damage through improving oxidative stress markers and regulating peroxidase activity pathways.

Chemical antioxidant activity reflects purely *in vitro* chemical radical-scavenging capacity and cannot be directly equated to in-vivo neuroprotective or memory-improving effects. Through relatively systematic *in vitro* assays for antioxidant activity, it was found that at concentrations of 0.05–0.40 mg/mL, Dragon’s Blood Capsules exhibited strong scavenging capacity against the 1,1-diphenyl-2-pyridylhydrazine (DPPḤ) radical, with a certain degree of concentration dependence (IC₅₀ = 0.14 mg/mL). Further studies revealed that Dragon’s Blood Capsules not only significantly scavenge O₂· (IC₅₀ = 0.24 mg/mL) and hydroxyl radicals (IC₅₀ = 0.057 mg/mL) generated by the NADH-PMS-NBT system, but also inhibit lipid peroxidation (IC₅₀ = 0.18 mg/mL), with a more pronounced effect than ascorbic acid (IC₅₀ = 0.27 mg/mL). In the β-carotene/linoleic acid autooxidation system, Dragon’s Blood Capsules demonstrated an antioxidant capacity of 37.8% at a concentration as low as 0.1 mg/mL (Shi and Yang, 2007). Multiple validation systems collectively confirm that Dragon’s Blood Capsules possess strong in-vitro radical-scavenging potential. Additionally, thin-layer chromatography combined with autoradiography was used to evaluate the antioxidant activity of the compounds based on the ratio of the peak area of the reference standard to the total peak area. The results showed the relative antioxidant contribution of different components: *Pterocarpus indicus*-derived constituents (25.69–42.72%), Loureirin B (6.83–19.34%), resveratrol (7.24–14.04%), and Loureirin A (5.50–14.20%) (Zhou et al., 2012). It should be emphasized that *Pterocarpus indicus* represents another botanical source of dragon’s-blood-type resin and is not derived from Dracaena cochinchinensis. Zhou et al. (2015) used the HPLC-DPPH method to evaluate the antioxidant capacity of active components in Dragon’s Blood. Pterostilbene (scavenging rate 73.19%) and resveratrol (scavenging rate 71.10%) showed comparable radical-scavenging activity. In the assay protecting human red-blood-cell membranes against lipid peroxidation, pterostilbene (IC₅₀ = 44.50 ± 7.80 μM) outperformed resveratrol (Mikstacka et al., 2010), while Loureirin B (39.01%) and Loureirin A (12.14%) exhibited lower scavenging rates than the two stilbene compounds. These in-vitro chemical assays suggest that stilbene-type compounds (e.g., pterostilbene, resveratrol) possess prominent chemical antioxidant activity, and antioxidant potency of dihydrocoumarin-type constituents can be enhanced by methoxy substitution forming symmetric structures; in contrast, 7,4′-dihydroxyflavones with hydroxyl groups at C-7 and C-4′ show negligible radical-scavenging effects. Therefore, stilbene and dihydrocoumarin derivatives are regarded as the major chemical antioxidant-active constituents of dragon’s-blood resin. Nevertheless, these above-mentioned observations are derived from chemical-level in-vitro systems and lack direct in-vivo cognitive-behavioral validation.

Cellular antioxidant signaling describes antioxidant responses observed in cultured cell models. At the cellular level, Loureirin B (10 μmol/L) markedly mitigates cell injury induced by oxygen–glucose deprivation/reoxygenation (OGD/R) in PC12 cells; this protective effect is mediated by activating the Nrf2 pathway and up-regulating HO-1 and Bcl-2 expression (Jiang et al., 2020). Moreover, certain dragon-blood-related substances can modulate autophagy-ROS balance in neuronal cells: increased LC3-II expression and autophagosome abundance were observed in H₂O₂-challenged spinal-cord neurons, accompanied by suppressed ROS production and relieved oxidative stress (He et al., 2017). These cellular findings demonstrate that dragon-blood-derived components can trigger intracellular antioxidant signaling cascades; however, these cell-based results cannot directly predict functional cognitive improvement in living animals.

Tissue-level neuroprotection and biochemical changes are reflected by altered oxidative-stress-related biomarkers in brain tissue from in-vivo cerebral-ischemia animal models. Both Dragon’s Blood dropping pills and Longxue Tongluo Capsules (LTC) (from Dracaena cochinchinensis) modulate oxidative-stress status in middle cerebral artery occlusion (MCAO) rats: they significantly elevate brain tissue levels of SOD, GSH-Px and CAT, regulate lactate dehydrogenase activity, and reduce MDA, iNOS, total-NOS and NO contents. Such biochemical remodeling strengthens cerebral antioxidant defense, ameliorates neurological deficits, and confers neuroprotection against focal cerebral ischaemia (Xin et al., 2013; Li and Luo, 2017). Stilbene-component-driven antioxidant effects involve modulation of cerebral antioxidant enzyme profiles and suppression of ischaemia-provoked oxidative damage (Yan et al., 2021; Lange and Li, 2018; Zhen et al., 2017). Of note, some publications investigated resin from *Pterocarpus indicus* rather than Dracaena cochinchinensis dragon’s-blood; in bilateral common-carotid-artery-occlusion reperfusion rat models, *Pterocarpus indicus* treatment restored CAT activity, lowered lipid peroxidation and nitrite levels, and mitigated oxidative injury, with mechanisms linked to NLRP3 inflammasome-gp91phox-related oxidative-stress pathways (Khan et al., 2021; Liu et al., 2017). For resveratrol, cerebral-ischaemia-reperfusion protection is tightly associated with antioxidant responses (Barbato et al., 2014; Wang et al., 2014). In MCAO rats, resveratrol nanoparticles increased GSH content and antioxidant-enzyme activities (GPx, GR, GST, SOD, CAT), decreased MDA concentration, and up-regulated Nrf2 and HO-1 protein levels, implying activation of the Nrf2/ARE antioxidant signaling axis (Ashafaq et al., 2021). Loureirin A can cross the BBB and distribute within rat brain tissue, enabling direct central-nervous-system-targeted action. In MCAO/R rats, Loureirin A treatment reduced brain MDA, enhanced SOD/CAT/GSH-Px activities and shrank cerebral infarct volume (Yang et al., 2010). Collectively, abundant in-vivo animal data confirm that dragon’s-blood-related interventions can reverse multiple oxidative-stress biochemical markers in ischaemic brain tissue. Importantly, most of these animal studies report neurological score and biochemical readouts, but do not simultaneously include dedicated behavioral-cognitive testing for memory function. Therefore, although antioxidant-biochemical and neuroprotective phenotypes are robust, causal inference linking these molecular changes to memory improvement remains preliminary without accompanying behavioral-cognitive evidence.

Behavioral cognitive outcomes represent the most direct endpoint for judging memory-ameliorating efficacy after cerebral ischaemia. Among the included literature, only a limited number of ischaemia-reperfusion animal studies combined antioxidant-biochemical detection with formal cognitive-behavioral assessment. For instance, resveratrol-nanoparticle pre-treatment reduced infarct volume and improved motor as well as cognitive performance in MCAO rats, together with restored cerebral antioxidant defense parameters (Cheng et al., 2015). Direct evidence that dragon’s-blood resin or its characteristic components (Loureirin A/B) can rescue ischaemia-induced memory impairment verified by Morris water maze, novel-object-recognition or equivalent cognitive behavioral assays is still relatively scarce.

In summary, Dragon’s Blood and its bioactive constituents modulate oxidative-stress responses across chemical, cellular and tissue levels. Stilbene and dihydrocoumarin compounds exhibit prominent *in-vitro* radical-scavenging capacity; in-vivo animal models demonstrate restoration of brain antioxidant enzyme systems via Nrf2/HO-1 and other signaling axes, accompanied by neuroprotective phenotypes. However, it should be noted that chemical antioxidant activity, cellular signaling activation, tissue-level biochemical improvement and behavioral cognitive recovery belong to distinct tiers of evidence. Biochemical antioxidant alterations alone cannot be simply extrapolated to definite memory-improving effects after cerebral ischaemia. Further experiments coupling oxidative-stress biomarker measurement with rigorous cognitive-behavioral evaluation are required to consolidate causal relationships.

#### 3.2.2 Inhibition of thrombosis and platelet aggregation

Thrombosis and abnormal platelet aggregation can obstruct blood supply to the brain, exacerbate ischemic damage to cognitive-related brain regions, and accelerate memory decline. Dragon’s Blood can inhibit platelet aggregation and improve cerebral microcirculation, thereby reducing the severity of cerebral ischemia at its source and creating favorable conditions for subsequently alleviating oxidative stress, restoring neural function, and relieving memory impairment.

The ethanol extract of Dragon’s Blood has been demonstrated to possess strong antithrombotic effects in thrombosis models in mice and rats. Its clinical antithrombotic effects are comparable to those of low-molecular-weight heparin, and it has shown significant efficacy in preventing deep vein thrombosis in patients undergoing hip fracture surgery (Xin et al., 2011; Liang et al., 2021). LTC is used to treat blood stasis syndrome during the recovery phase of cerebral infarction. No significant toxicity was observed in 90-day long-term toxicity studies conducted on rats and Beagles, and clinically, it has been shown to effectively improve neurological deficits and disability during the recovery phase of stroke (Zhao et al., 2016; Dai et al., 2026). Another study investigated the clinical efficacy of combining LTC with Liver-Soothing and Depression-Relieving Capsules and Vinpocetine for the treatment of thrombotic cerebral infarction during the recovery phase. The study found that this combination promotes cerebral blood circulation, alleviates patients’ clinical symptoms, and thereby accelerates the recovery process. The clinical treatment efficacy rate reached as high as 93.06%, with no significant side effects, making the treatment safe and reliable (Wang et al., 2019; Zhu, 2020). In addition, Liang et al. (2016) found that Dragon’s Blood total phenolic extract pills significantly increased cerebral blood flow in anesthetized dogs; at a dose of 35 mg/kg, they significantly reduced cerebral vascular resistance within 1 h, improved cerebral ischemia, and restored neurological function following cerebral infarction. Abnormal expression of plasminogen activator inhibitor-1 (PAI-1) can disrupt the body’s coagulation and anticoagulation systems, leading to thrombus formation (Griemert et al., 2019). In areas of cerebral ischemia, PAI-1 is induced and plasma tPA levels are reduced, thereby promoting fibrin clot formation and exacerbating brain damage (Tuttolomondo et al., 2009). Studies have confirmed that Loureirin B acts as a PAI-1 inhibitor (Jiang et al., 2017) and can significantly increase the rate of fibrinolysis, exhibiting a dose–response relationship with the inhibition of PAI-1/uPA complex formation. In a FeCl₃-induced murine thrombosis model, treatment with Loureirin B prolonged bleeding time to 50–90% of the original value, significantly reduced thrombus weight, and markedly decreased the area of intravascular thrombus formation, demonstrating a pronounced antithrombotic effect.

LTC inhibits adenosine diphosphate/platelet-activating factor-induced platelet aggregation in rats and rabbits *in vitro*, improves the hemorheological properties of blood in rats with blood stasis, and exerts a significant protective effect against brain tissue damage following focal cerebral ischemia (Zhao et al., 2016). Loureirin A can inhibit thrombus formation in rats both *in vivo* and *in vitro*, prolong the *in vitro* clotting time in rats, and reduce whole blood viscosity, hematocrit, and fibrinogen levels; its mechanism of action is related to the inhibition of the PI3K/AKT signaling pathway in platelets (Xin et al., 2011; Sang, 2015). Recent studies have found that Loureirin A inhibits the activity of collagen, thrombin, and adenosine diphosphate (ADP), thereby inhibiting platelet aggregation; its antiplatelet activity and mechanisms for improving blood flow are associated with the inhibition of Akt phosphorylation at the Ser473 site (Hao et al., 2015). Using thrombin as a receptor, it was found that at various concentrations, the *in vitro* thrombin activity of Loureirin A was higher than that of Loureirin B (Liu et al., 2015), suggesting that both Loureirin A and Loureirin B exert strong inhibitory effects on platelet aggregation and can, to varying degrees, alleviate thrombus formation and platelet aggregation caused by cerebral ischemia.

#### 3.2.3 Anti-inflammation

Cerebral ischemia triggers both central and peripheral inflammatory responses, which are central pathological mechanisms mediating neuronal apoptosis, synaptic damage, cognitive decline, and memory impairment. Dragon’s Blood exhibits significant anti-inflammatory effects and plays an important role in suppressing inflammation in organs and tissues such as the brain, heart, intestines, liver, and lungs (Zhang X. et al., 2022; Li et al., 2018; Zhong et al., 2018). Studies have found that Dragon’s Blood can reduce serum levels of tumor necrosis factor-α (TNF-α) and interleukin (IL)-6 in rats with traumatic brain injury, thereby alleviating the inflammatory response and exerting a protective effect against traumatic brain injury (Wang and Li, 2012). The ability of Dragon’s Blood to reduce TNF-α and IL-6 levels has also been confirmed in studies on anti-inflammatory effects in rats with radiation-induced brain injury, where it alleviates radiation-induced neuroinflammation exacerbated by endogenous neurotoxins (Xin et al., 2012; Zhang et al., 2020).

During cerebral ischemia, overactivated astrocytes release various pro-inflammatory factors, which enhance the neuroinflammatory response and exacerbate brain tissue damage (Zhang et al., 2018; Wu et al., 2022); therefore, specifically inhibiting astrocyte activation can serve as an effective means of reducing the inflammatory response and further alleviating brain tissue damage. *In vivo* studies have shown that resveratrol can significantly inhibit astrocyte activation in a rat model of MCAO, thereby reducing brain damage and improving neurological function (Yu et al., 2017). *In vitro* studies have also reported (Liu et al., 2020) that 24-h pretreatment with resveratrol can inhibit astrocyte proliferation and activation following OGD/R, thereby reducing the expression of pro-inflammatory factors TNF-α and IL-1β while enhancing the expression of anti-inflammatory factors IL-10 and IFN-β, thus alleviating the inflammatory response. In the inflammatory response following I/R, nuclear factor κB (NF-κB) can induce the production of pro-inflammatory factors by activating the NLRP3 inflammasome and astrocytes. Selective inhibition of NF-κB can reduce astrocyte activation and may serve as an effective strategy for reducing inflammatory damage to the central nervous system (Guan et al., 2013; Yuan et al., 2017).

In both the MCAO/R mouse model and the OGD/R-treated HT22/U251 co-culture model, it was found that Pteris sinensis reduced the production of TNF-α, IL-1β, IL-6, cyclooxygenase-2 (COX-2), iNOS, histamine, prostaglandin E2, and other inflammatory cytokines, as well as downregulate the expression of matrix metalloproteinases (MMPs), such as MMP-2 and MMP-9. Further mechanistic studies revealed that this effect is achieved by inhibiting NF-κB phosphorylation, thereby alleviating the inflammatory response induced by astrocyte activation following I/R injury, and consequently exerting an anti-inflammatory effect on cerebral I/R injury (Liu et al., 2019; Viana et al., 2020).

Microglia in the brain play a key role in neuroinflammation associated with ischemic brain injury. Cerebral ischemia leads to the overexpression of inflammatory factors in hyperactivated microglia, which in turn increases the extent of cerebral infarction and exacerbates brain injury. Studies have shown that both the ethanol extract of Dragon’s Blood and LTC can significantly reduce the release of relevant inflammatory factors (IL-1β, IL-6, NO), exerting anti-inflammatory effects that improve brain injury. The mechanism underlying this effect is related to the regulation of lipopolysaccharide (LPS)-induced COX-2 gene expression, which inhibits microglial activation (Li et al., 2014; Heo et al., 2010; Yuan, 2016; Choy et al., 2008; Sun et al., 2019a). The activation of microglia can be classified into two distinct phenotypes: M1 and M2. The M1 phenotype has a pro-inflammatory effect that exacerbates brain injury, while the M2 phenotype has an anti-inflammatory effect and participates in the repair and remodeling of damaged tissue (Li et al., 2025b). Studies have confirmed that microRNA-155 (miR-155) promotes M1 polarization of microglia, thereby increasing neuroinflammatory responses. An MCAO model was established in C57/BL mice, and resveratrol (100 mg/kg) was administered intraperitoneally at 0, 8, and 18 h after ischemia. It was found that resveratrol reduced miR-155 expression, thereby inhibiting M1 polarization of microglia, decreasing the expression of M1-specific genes CD32 and IL-1β as well as the levels of IL-1 and IL-6, promoting M2 polarization of microglia, significantly increasing the expression of M2-specific genes CD206 and ARG-1 and the levels of IL-4 and IL-10, and effectively reducing neuroinflammation following cerebral ischemia (Ma et al., 2020). Other studies have also shown that administration of resveratrol (10^−^7 mol/L) to MCAO mice inhibits the excessive activation of microglia and reduces the release of inflammatory cytokines (IL-1β, IL-6, TNF-α), thereby alleviating the inflammatory response associated with ischemic brain injury. Further mechanistic studies suggest that this effect may be related to the downregulation of the CD147/MMP-9 pathway and the regulation of imbalances in the expression of BDNF/Akt/CREB and TREM1/2 (Xu et al., 2021; Zhang and Zhao, 2022). Further mechanistic studies suggest that this effect may be related to the downregulation of the CD147/MMP-9 pathway and the regulation of imbalances in the expression of BDNF/Akt/CREB and TREM1/2 (Jiang et al., 2020; Tang et al., 2019).

A recent study first isolated a novel cyclic dihydrochalcane trimer, nordracophane (alongside dracophane), from the resin of *Dracaena cochinchinensis* (Chinese Dragon’s Blood). Nordracophane effectively inhibits LPS-induced NO release from RAW264.7 macrophages with an IC₅₀ of 14.9 ± 4.5 μmol/L, demonstrating notable anti-inflammatory activity (Pang et al., 2019).

The active components of Dragon’s Blood—santalol and resveratrol—both exert anti-inflammatory effects by inhibiting the activation of astrocytes and microglia, thereby reducing the release of related inflammatory factors. Furthermore, existing mechanistic studies suggest that the protective effect of Dragon’s Blood against I/R injury may be associated with the inhibition of cell activation, NF-κB phosphorylation, and NLRP3 inflammasome activation. Dragon’s Blood may still contain other potential novel anti-inflammatory compounds; further investigation of these compounds will aid in the development of new anti-inflammatory drugs and hold significant implications for the treatment of inflammatory responses associated with I/R injury.

#### 3.2.4 Protection of the blood–brain barrier

Following ischemia, a massive release of inflammatory factors and an abnormal increase in vascular permeability occur, leading to structural damage to the BBB and further inducing vasogenic cerebral edema. Cerebral edema can compress neural tissue, damage memory-related brain regions such as the hippocampus, and disrupt synaptic plasticity; it is a major pathological factor contributing to cognitive impairment and memory deficits. Impaired BBB integrity is a key early event in I/R injury. Cerebral I/R injury disrupts BBB integrity, thereby impairing normal brain function, and this disruption of BBB function and structure can exacerbate the progression of cerebral I/R injury (Liebner et al., 2018).

Cerebral ischemia causes increased permeability of the BBB, leading to the extravasation of blood components and macromolecules, which in turn results in hemorrhagic edema and hemorrhagic transformation, potentially causing irreversible secondary damage (Brouns and De Deyn, 2009). BBB permeability has become a key therapeutic target for cerebral ischemia. Previous studies have shown that in rats with an MCAO model, after 14 days of continuous administration of Dragon’s Blood Resin, the high-dose group (2.60 g/kg) exhibited a significant reduction in both cerebral infarct volume (32.42% ± 2.57) and brain tissue water content (70.31% ± 1.15), with the best recovery from neurological damage. Both Dragon’s Blood Pellet and LTC significantly reduced brain water content in rats with focal cerebral ischemia, and the effect showed a dose-dependent trend (Xin et al., 2013; Li and Luo, 2017).

Resveratrol plays a beneficial role in maintaining BBB function (Lei et al., 2019). In a rat model of cerebral ischemia, intraperitoneal injection of resveratrol (100 mg/kg) 24 h after the ischemic event significantly reduced the activity of myeloperoxidase—a marker of macromolecular permeability—and the concentration of Evans Blue (EB), while also significantly reducing the water content in brain tissue on the ischemic side. Another study using the wet-dry weight method confirmed that even a lower dose of resveratrol (40 mg/kg) could significantly reduce brain tissue water content (77.65% ± 4.02) in rats with a MCAO model (Zhang et al., 2021).

Further studies have shown that resveratrol can inhibit bacterial penetration and reduce BBB permeability by suppressing the ERK1/2-VEGFA signaling cascade and attenuating the upregulation of caveolin-1 (CAV-1). It significantly inhibits the invasion of the BBB by *Escherichia coli* causing meningitis and downregulates ICAM-1 and VCAM-1, thereby improving BBB permeability and ultrastructure in MCAO rats and reducing the severity of cerebral edema following I/R (Zhang et al., 2021; Yang et al., 2021). Other active compounds also demonstrated a significant ability to improve BBB permeability. In a mouse model of cerebral IR, continuous administration of Pteris sinensis for 5 days resulted in a dose-dependent reduction in both cerebral edema and Na^+^ concentration, with the most pronounced effect observed at a dose of 10 mg/kg (Yang et al., 2010). The above studies indicate that Dragon’s Blood and its bioactive compounds can protect brain tissue by reducing the permeability of the BBB, thereby lowering the water content in brain tissue and alleviating cerebral edema (Zhang X. et al., 2022).

As the fundamental structural units and primary components of the BBB, vascular endothelial cells play a crucial role in maintaining BBB integrity and represent a key target for the treatment of cerebral ischemia. Studies have shown that phenolic extracts from Dragon’s Blood can alleviate endothelial dysfunction; at a concentration of 10 mg/L, LTC can increase the survival rate of HUVECs following OGD/R injury and effectively protect endothelial cells in hypoxic conditions (Sun et al., 2019a). In addition, it exerts a significant protective effect against HUVEC damage induced by oxidized low-density lipoprotein, and the mechanism was found to be related to the inhibition of OGD/R-induced activation of the mitochondrial-associated caspase-3/9 pathway (Pan et al., 2018; Wang et al., 2018). Administration of the active ingredient resveratrol (40 mg/kg) played a certain role in restoring the ultrastructure of the BBB in rats with a MCAO model; the cellular structures of the BBB components—including astrocytes and endothelial cells—remained relatively intact, and the extent of damage was milder than in the control group (Zhang et al., 2021). Existing research has confirmed that resveratrol’s effects on vascular endothelial cells can effectively activate the Wnt/β-catenin signaling pathway, increase the expression of claudin-5 and tight junction proteins, and reduce the permeability of the BBB, thereby reversing damage to the BBB (Shi et al., 2020). I/R causes endothelial cell damage, leading to endothelial-to-mesenchymal transition. Studies have shown that Pteris sinensis (10 mg/kg) can significantly inhibit excessive endothelial-to-mesenchymal transition in MCAO rats; its mechanism of action may be related to the activation of the Akt/GSK-3β pathway (Wang et al., 2022). The basement membrane, which connects endothelial cells, is another important barrier of the BBB. Both the extracellular matrix and the vascular basement membrane—key structures that maintain BBB integrity—serve as substrates for MMPs. During cerebral ischemia, elevated MMP-9 expression can compromise BBB integrity, leading to BBB disruption and vasogenic cerebral edema. Following I/R injury, a significant increase in MMP-9 expression leads to the degradation of tight junction proteins, increases microvascular permeability, and consequently causes cerebral edema. Resveratrol inhibits MMP-9 activity and increases TIMP-1 activity to maintain BBB integrity, thereby reducing cerebral edema and alleviating cerebral I/R injury (Wei et al., 2015).

Dragon’s Blood has a protective effect on the integrity of the BBB following cerebral ischemia. It works by alleviating endothelial dysfunction and protecting the vascular basement membrane, thereby jointly maintaining the permeability and integrity of the BBB. Among its active compounds, resveratrol exhibits a particularly strong protective effect on the BBB; it maintains the structural integrity of the cells comprising the BBB, demonstrates outstanding efficacy in combating cerebral edema, and exerts a potent anti-ischemic effect.

## 4 Conclusion and future perspectives

Due to its multi-target pharmacological properties observed in preclinical investigations, Dragon’s Blood has garnered significant attention in preclinical intervention studies on cerebral ischemia and secondary cognitive impairment. Its primary active constituents are phenolic compounds; active monomers including pterostilbene, resveratrol, and Loureirin B constitute the key molecular basis underlying its neuroprotective effects. Cumulative preclinical evidence demonstrates that Dragon’s Blood and its bioactive components can interfere with the pathological progression of cerebral ischemia via multiple pharmacological mechanisms - including anti-oxidative-stress activity, neuroinflammation inhibition, blood–brain-barrier protection, anti-thrombotic action, and neurotrophic effects - and thus ameliorate neuronal injury and memory deficits induced by cerebral ischemia (Figure 3). Modern studies have revealed that cerebral ischaemic injury manifests staged pathological features with overlapping mechanisms. Inflammation, dysregulated oxidative stress, microcirculatory disturbance, neuronal programmed cell death, and aberrant angiogenesis represent core targets contributing to post-ischaemic cognitive and memory impairment (Lu et al., 2026; Chen W. et al., 2026; Jia et al., 2025; Fang et al., 2024). These multidimensional, multi-stage pathogenic features match the multi-target, holistic action of Dragon’s Blood (Resina Draconis), supporting its value in post-ischemic memory impairment. Given complementary mechanisms, co-administration with edaravone, vinpocetine, nimodipine or donepezil may yield synergistic benefits, though direct evidence is limited and warrants further preclinical validation (Zhu, 2020). While improving cerebral perfusion, protecting neural function, and enhancing learning and memory abilities, it can reduce the dosage of Western medications used alone and lower the risk of adverse reactions, fully demonstrating the therapeutic characteristics and practical value of integrated Traditional Chinese and Western medicine - specifically, the TCM principles of promoting blood circulation, resolving blood stasis, unblocking meridians, and enhancing cognitive function (Lu et al., 2024; Xu, 2022). In summary, cumulative preclinical evidence indicates that Dragon’s Blood and its bioactive components can target multiple pathological stages of cerebral ischaemic injury and ameliorate associated cognitive and memory deficits, suggesting promising research value. However, its clinical efficacy and application prospects for cerebral-ischaemia-related cognitive impairment remain to be confirmed by further high-quality clinical trials.

**Figure 3:**
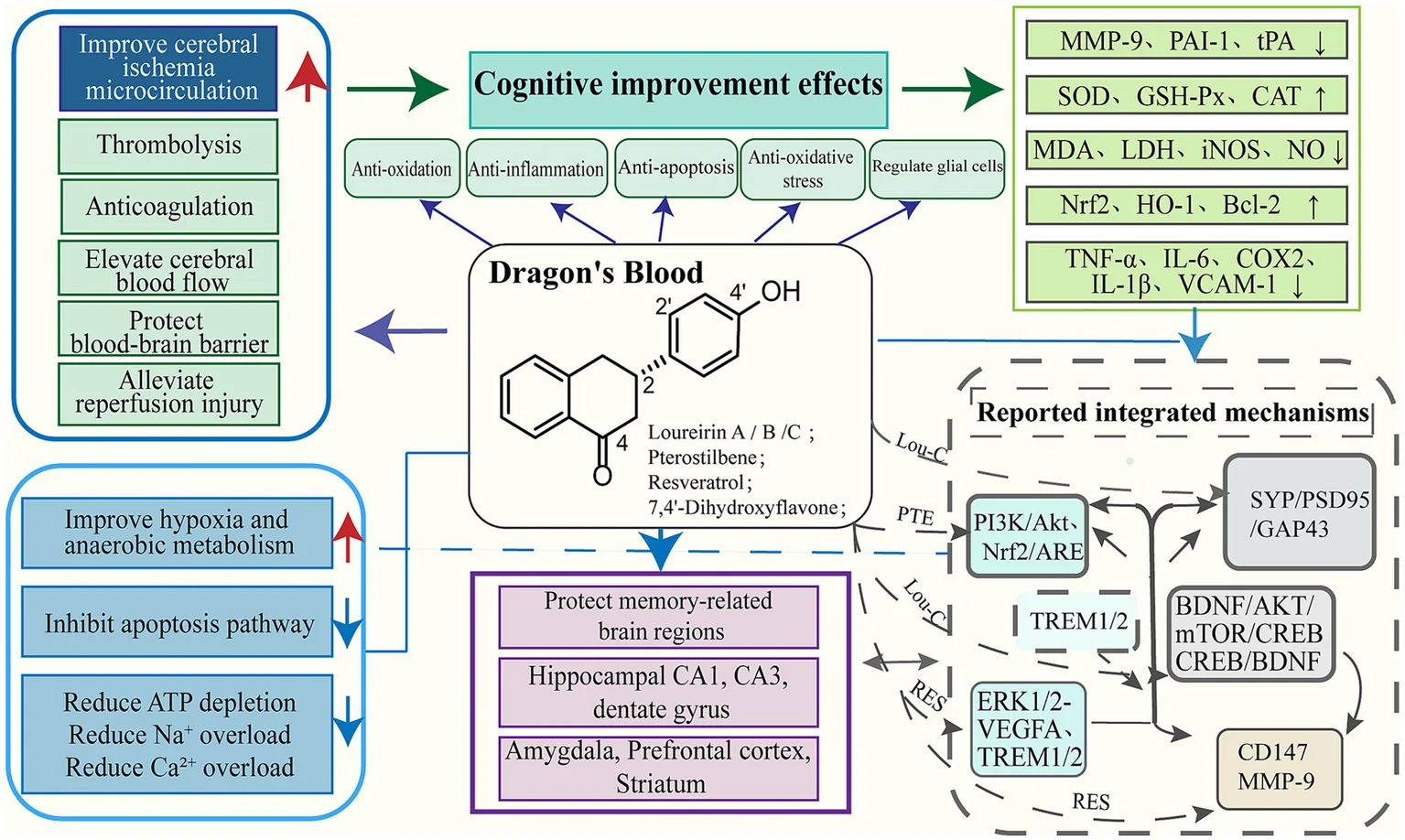
Mechanism of dragon’s blood in alleviating memory impairment after cerebral ischemia. Solid lines indicate direct regulatory effects; dashed lines represent reported indirect molecular-pathway associations. ↑, increased molecular level; ↓, decreased molecular level. BBB, blood–brain barrier; GSH-Px, glutathione peroxidase; MDA, malondialdehyde; LDH, lactate dehydrogenase; iNOS, inducible nitric oxide synthase; COX2, cyclooxygenase-2; VCAM-1, vascular cell adhesion molecule-1; PI3K, phosphoinositide 3-kinase; Akt, protein kinase B; Nrf2, nuclear factor erythroid 2-related factor 2; ARE, antioxidant response element; mTOR, mammalian target of rapamycin; CREB, cAMP response element-binding protein; BDNF, brain-derived neurotrophic factor; VEGFA, vascular endothelial growth factor A; TREM1, triggering receptor expressed on myeloid cells 1; TREM2, triggering receptor expressed on myeloid cells 2.

At present, research on the pharmacological effects and mechanisms of Dragon’s Blood resin against cerebral ischemia and post-ischemic memory impairment has gradually advanced. Nevertheless, multiple limitations remain to be addressed in this field. Existing pre-clinical studies mainly focus on exploring neuroprotective mechanisms of individual Dragon’s Blood monomers via specific signaling pathways and molecular targets, with an emphasis on verifying their correlations with cerebral ischemic injury and cognitive dysfunction. However, these studies have largely neglected horizontal investigations into the cross-talk and synergistic regulation among multiple signaling pathways and targets. The core pharmacological effects of Dragon’s Blood - including anti-oxidative-stress activity, anti-inflammation, alleviation of cerebral ischaemia-reperfusion injury, and neural-function protection - arise from the synergistic action of its multiple bioactive components. Consequently, existing studies struggle to fully elucidate the scientific basis underlying its overall pharmacological effects. Meanwhile, the potential of numerous active monomers in Dragon’s Blood against cerebral ischaemia and cognitive impairment remains to be explored, and the molecular regulatory mechanisms of known core components require further detailed investigation.

At the experimental model level, current research on the anti-cerebral-ischaemia and memory-improving effects of Dragon’s Blood largely depends on limited basic experimental models, leading to insufficient generalizability and poor clinical relevance. In the future, a more comprehensive and diverse system of *in vivo* and *in vitro* cerebral ischaemia-injury models should be established. Transgenic animals, gerbils, rabbits, minipigs and cynomolgus macaques, which possess high similarities to human brain structure, can be adopted to systematically dissect the full mechanism through which Dragon’s Blood-derived active components modulate pathological progression of cerebral ischaemia and ameliorate cognitive-memory deficits. Combined with cellular-level experiments for thorough validation of core molecular mechanisms, these multi-model studies may help precisely clarify the integrated mechanism underlying Dragon’s Blood-mediated protection against cerebral ischaemia and ischaemia-induced memory impairment, and further consolidate the experimental and theoretical basis for its subsequent clinical-translational research.

Current clinical observations suggest that Dragon’s Blood resin exhibits good tolerability with rare adverse reactions in routine cerebrovascular disease treatment. However, relevant clinical evidence is mostly derived from general cardiovascular and cerebrovascular studies, and high-quality clinical data specifically focusing on post-ischemic cognitive and memory protection remain insufficient. Preclinical animal studies have demonstrated that total phenol drops of Dragon’s Blood can effectively improve cerebral microcirculation by increasing cerebral blood flow and reducing cerebral vascular resistance, thereby maintaining normal cerebral perfusion. These findings provide solid experimental support for its microcirculation-regulating, neuroprotective, and cognitive-improving effects under ischemic conditions (Jiang et al., 2026; He et al., 2024). Compared to the widespread use of Dragon’s Blood in treating multisystem diseases such as anorectal disorders (Fan et al., 2014), traumatic swelling and pain (Li et al., 2025a), and pancreatic injury (Tan et al., 2026), to date, dedicated clinical research focusing on Dragon’s Blood for the treatment of cerebral ischemia and subsequent memory impairment remains relatively limited. Clinical observations have demonstrated that cognitive dysfunction following cerebral ischemia presents obvious regional selectivity in brain damage. Therefore, future therapeutic strategies targeting post-ischemic memory improvement should focus on the targeted protection of core memory-related brain regions, including the hippocampus, prefrontal cortex, and thalamus, so as to alleviate neuronal injury and neural circuit disruption induced by ischemic insult. Although the striatum also participates in memory-cognitive processing, direct in-vivo evidence for Dragon’s Blood-mediated protection against ischemic striatal injury is currently absent, and this potential effect requires further experimental validation. Future work should build on comprehensive basic mechanistic research to explore its multi-target therapeutic advantages and the value of integrated traditional Chinese and Western medicine; however, its real-world clinical application for ischemic cerebrovascular disease and cognitive impairment cannot be overstated without adequate clinical evidence.

From a clinical translational perspective, current evidence for Dragon’s Blood is largely derived from small-sample, single-center observational studies in ischemic-stroke patients, predominantly using oral capsule or dropping-pill preparations. These trials mainly evaluate neurological deficit scores, cerebral blood flow and activities of daily living, and are limited by non-uniform dosage regimens and inconsistent follow-up durations. It is therefore necessary to distinguish general cerebrovascular protective evidence from evidence specifically supporting post-ischemic cognitive-memory recovery: existing clinical data support its use in stroke rehabilitation, but well-designed trials with cognitive function (e.g., MoCA, MMSE) as the primary endpoint remain scarce, and its efficacy for cognitive recovery cannot be directly extrapolated from general stroke outcomes. Although severe adverse events are rarely reported in routine practice, large-sample safety data specifically for patients with ischemic cognitive impairment are still insufficient. Accordingly, the clinical value of Dragon’s Blood for cerebral-ischemia-related memory impairment remains to be confirmed by further multicenter, well-controlled trials with cognitive-focused primary endpoints.
